# Deoxyhypusine synthase deficiency syndrome zebrafish model: aberrant morphology, epileptiform activity, and reduced arborization of inhibitory interneurons

**DOI:** 10.1186/s13041-024-01139-w

**Published:** 2024-09-27

**Authors:** Elham Shojaeinia, Teresa L. Mastracci, Remon Soliman, Orrin Devinsky, Camila V. Esguerra, Alexander D. Crawford

**Affiliations:** 1 Center for Molecular Medicine Norway (NCMM), University of Oslo, Oslo, Norway; 2 Institute for Orphan Drug Discovery, Bremerhaven, Germany; 3 Department of Biology, Indiana University-Indianapolis, Indianapolis, IN USA; 4 Luxembourg Centre for Systems Biomedicine (LCSB), University of Luxembourg, Belvaux, Luxembourg; 5 Department of Neurology, New York University Langone Medical Center, New York, NY USA

**Keywords:** Zebrafish, Deoxyhypusine synthase, Hypusine, Epilepsy, Neurodevelopmental disorder

## Abstract

DHPS deficiency syndrome is an ultra-rare neurodevelopmental disorder (NDD) which results from biallelic mutations in the gene encoding the enzyme deoxyhypusine synthase (*DHPS*). DHPS is essential to synthesize hypusine, a rare amino acid formed by post-translational modification of a conserved lysine in eukaryotic initiation factor 5 A (eIF5A). DHPS deficiency syndrome causes epilepsy, cognitive and motor impairments, and mild facial dysmorphology. In mice, a brain-specific genetic deletion of *Dhps* at birth impairs eIF5A^HYP^-dependent mRNA translation. This alters expression of proteins required for neuronal development and function, and phenotypically models features of human DHPS deficiency. We studied the role of DHPS in early brain development using a zebrafish loss-of-function model generated by knockdown of *dhps* expression with an antisense morpholino oligomer (MO) targeting the exon 2/intron 2 (E2I2) splice site of the *dhps* pre-mRNA. *dhps* knockdown embryos exhibited dose-dependent developmental delay and dysmorphology, including microcephaly, axis truncation, and body curvature. In *dhps* knockdown larvae, electrophysiological analysis showed increased epileptiform activity, and confocal microscopy analysis revealed reduced arborisation of GABAergic neurons. Our findings confirm that hypusination of eIF5A by DHPS is needed for early brain development, and zebrafish with an antisense knockdown of *dhps* model features of DHPS deficiency syndrome.

## Introduction

Post-translational modification is a core cellular strategy to rapidly alter protein activity in response to environmental stimuli. Over 200 post-translational modifications are mediated by enzymes which facilitate diverse reactions including phosphorylation, ubiquitination, glycosylation, palmitoylation, sulfation, methylation, small ubiquitin-like modifier (SUMO)-ylation, and nitrosylation, among others. A unique post-translational modification is hypusination, limited to eukaryotic translation factor 5 A (eIF5A) proteins [1]. Hypusination enables eIF5A activation via enzymatic conversion of a conserved lysine to the novel amino acid hypusine (N^ϵ^-4-amino-2-hydroxybutyl(lysine)). This reaction occurs in two steps: (1) lysine residue modification by deoxyhypusine synthase (DHPS), using the polyamine spermidine as a cofactor, to form the intermediate deoxyhypusine; (2) hydroxylation of this residue by deoxyhypusine hydroxylase (DOHH) in an oxygen-dependent reaction [2] to form hypusine.

Hypusinated eIF5A (eIF5A^HYP^) has key cellular functions: (1) facilitating initiation, elongation or termination during the translation of cell type-specific transcripts [1, 3–6]; (2) suppressing ribosomal stalling by stabilizing tRNA-ribosomal P-site interaction, facilitating peptide bond formation for consecutive polyprolines and other tripeptide motifs [7, 8]; and (3) nonsense-mediated decay (NMD) of mRNA transcripts with premature stop codons [9]. eIF5A^HYP^ is critical to translate long polypeptides [10].

eIF5A, as well as the DHPS and DOHH enzymes required for activation by hypusination, are essential for eukaryotic cell viability and growth. In yeast, eIF5A^HYP^ controls cell proliferation and is required for polarized cell growth during mating by regulating the translation of polyproline-rich formins [11]. In plants, eIF5A activation by hypusination is essential for growth during development and for environmental stress responses [12].

The DHPS-DOHH-eIF5A pathway relies on an oxygen-sensing mechanism. In yeast, DOHH deficiency impairs hydroxylation of the deoxyhypusine residue in eIF5A, decreasing N-terminal translation of proteins in mitochondrial respiration, oxidative stress response, and protein folding. eIF5A hypusination adapts cellular metabolism to oxygen levels [2].

In mammals, the DHPS-DOHH-eIF5A pathway is implicated in disease pathogenesis and in aging. In the *Drosophila* brain, hypusinated eIF5A levels decline with age, but can be increased by dietary spermidine, and genetic attenuation of eIF5A^HYP^ levels induces premature aging (e.g., reduced mitochondrial respiration) [13]. Spermidine supplementation in mice boosts eIF5A hypusination and improves cognitive function [14].

Mice with T-cell-specific deletions of *Dohh* and *Dhps* develop severe intestinal inflammatory disease, supporting the role of hypusination in T cell activation and differentiation, long associated with polyamine synthesis [15]. Conversely, mice with a myeloid-specific deletion of *Dhps* revealed that eIF5A^HYP^ promotes a pro-inflammatory macrophage M1-like phenotype [16].

Cancer-related signaling pathways regulated by eIF5A^HYP^ include MYC, p53, and hypoxia-inducible factor 1-alpha (HIF1A). Overexpression of eIF5A is linked to colorectal, gastric, esophageal, lung, breast, ovarian, cervical, bladder, prostate, and hepatocellular cancers. Therefore, small-molecule inhibitors of hypusination that target DHPS or DOHH are potential anti-neoplastics [17].

In humans, mutations in the DHPS-DOHH-eIF5A pathway cause neurodevelopmental disorders. A rare, autosomal dominant disorder caused by heterozygous pathogenic EIF5A variants results in developmental delay, intellectual disability, microcephaly, and facial dysmorphism [18]. Rare, autosomal recessive biallelic pathogenic missense and truncating DOHH variants cause developmental delay, intellectual disability, microcephaly, facial dysmorphism, and epilepsy [19]. DHPS deficiency causes an ultra-rare, autosomal recessive disorder caused by biallelic pathogenic variants that reduce DHPS enzyme activity (~ 18–25% of normal), with features similar to the eIF5A and DOHH deficiency syndromes [20].

Homozygous knockout mouse models of DHPS, DOHH and eIF5A are early embryonic lethal, underscoring the essential role of this pathway in early development [21–23]. In mice, conditional knockout mouse models of these genes support their developmental roles. Mice with conditional genetic deletions of *Dhps* or *Eif5a* induced by the Emx1-Cre driver (primarily expressed in the cortex and hippocampus from E9.5 onwards) show gross defects in forebrain development, reduced growth, and premature death [24]. Mice with a brain-specific deletion of *Dhps* initiated at birth (via intraventricular injection of an adeno-associated virus with CMV-driven Cre expression) exhibited spontaneous seizures, impaired growth, and death before 6 weeks of age. Moreover, proteomic analysis of brain tissue using quantitative mass spectrometry revealed that these brain-specific *Dhps* knockout mice had changes in numerous proteins involved in neuronal growth, function, and secretion [3].

In patients and animal models, the DHPS-DOHH-eIF5A pathway disorders primarily impact the brain. While conditional mouse knockouts provide valuable data on the role of eIF5A^HYP^ in postnatal brain development and function, zebrafish models can probe the role of hypusination in early brain development. A zebrafish model for eIF5A deficiency revealed mild microcephaly and micrognathia [18, 25]. A zebrafish model for DHPS deficiency focused on pancreas development [26]. Here, we characterize a zebrafish DHPS deficiency model, assessing the impact of reduced *dhps* expression on early brain development and activity.

## Results

### Generation of a zebrafish model for DHPS deficiency

**Fig. 1:**
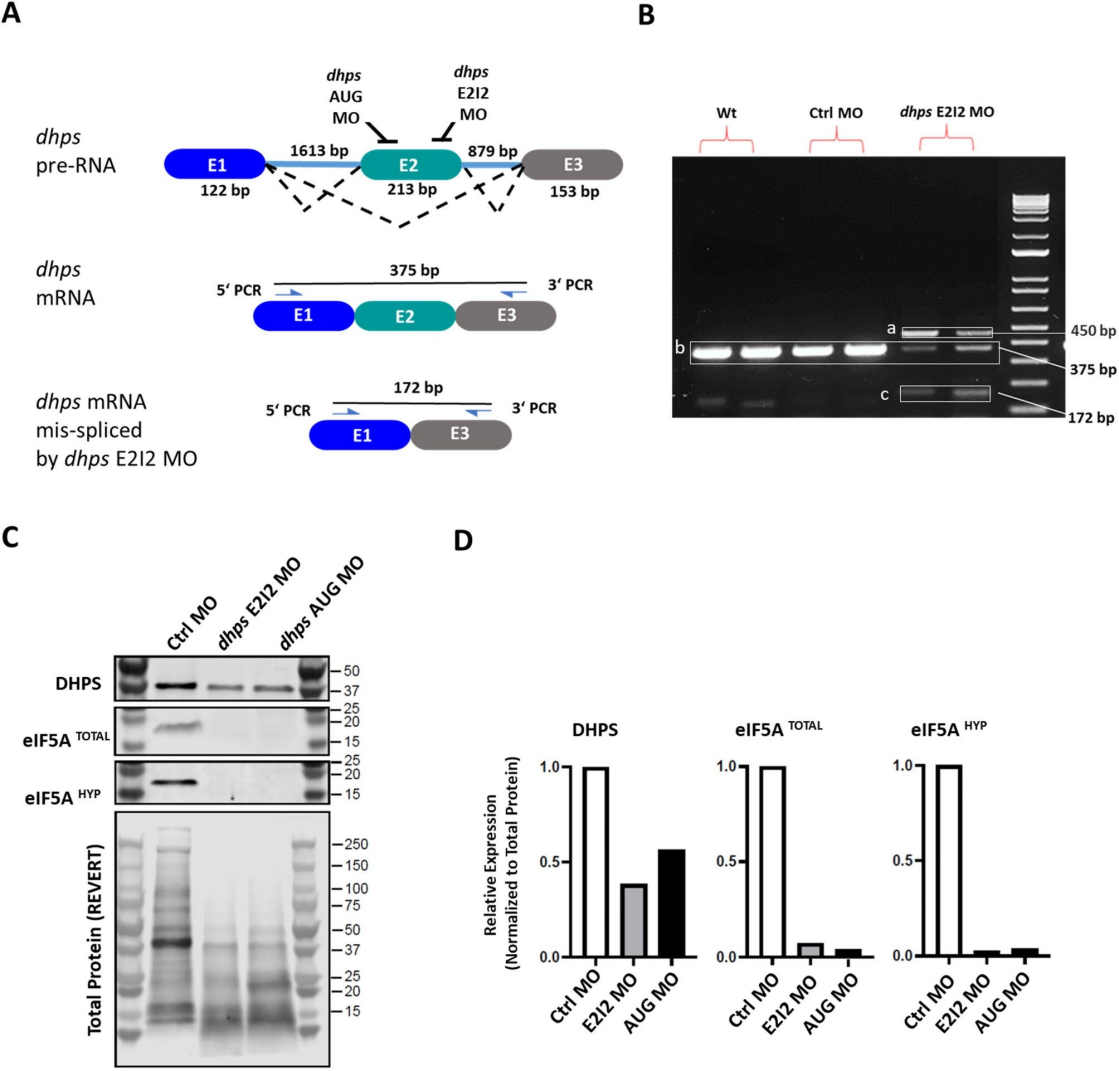
Generation of the DHPS deficiency zebrafish model. (**A**) The first three exons and introns of the *dhps* gene and selected targeting sites of two antisense morpholino oligomers and PCR primers are shown, as well as the predicted amplicon lengths. (**B**) RT-PCR analysis confirmed the predicted lengths of the *dhps* mRNA amplicons from Wt, Ctrl MO and *dhps* E2I2 MO larvae with two biological repeats. While the length of the PCR amplicon covering the first three exons of *dhps* mRNA is 375 bp (b) in Wt, Ctrl MO, and *dhps* E2I2 MO larvae, in the latter two, additional bands appear (a and c). Appearance of the 172-bp amplicon (c), indicating deletion of exon 2 from the *dhps* mRNA, and reduction in amount of the 375-bp amplicon (b), together confirm partial knockdown of *dhps*. Appearance of an approximately 450-bp amplicon (a) in *dhps* E2I2 MO larvae may be due to partial activation of a cryptic splice site induced by antisense blockage of the E2I2 splice site. (**C**) Analysis of protein expression in *dhps* knockdown zebrafish. Zebrafish embryos (2 dpf) with knockdown by *dhps* E2I2 MO or *dhps* AUG MO were analyzed by Western blot for expression of DHPS, eIF5A^TOTAL^, eIF5A^HYP^, and total protein (as visualized by REVERT™). (**D**) Densitometric data for the relative expression of DHPS, eIF5A^TOTAL^, and eIF5A^HYP^, normalized on the basis of total protein as quantified by REVERT™ staining

Antisense morpholino oligomers (MOs) were designed targeting the AUG start codon of the zebrafish *dhps* mRNA (*dhps* AUG MO), and the E2I2 splice site of the zebrafish *dhps* pre-mRNA (*dhps* E2I2 MO) (Fig. 1A). Knockdown efficacy of the *dhps* E2I2 MO was analyzed by reverse transcriptase PCR (RT-PCR), which confirmed aberrant splicing (loss of exon 2) after microinjection of the *dhps* E2I2 MO, resulting in the appearance of a 172-bp amplicon and reduction of the 375-bp amplicon versus the control morpholino (Ctrl MO) and uninjected wild-type (Wt) larvae (Fig. 1B). Western blot analysis revealed a reduction in dhps protein levels in knockdown embryos generated using the *dhps* AUG MO and the *dhps* E212 MO. *dhps* knockdown embryos also had strongly reduced levels of both eif5a^HYP^ and eif5a^TOTAL^, with a striking reduction of larger polypeptides (Fig. 1C and D).

### Phenotypic analysis of *dhps* knockdown zebrafish

**Fig. 2:**
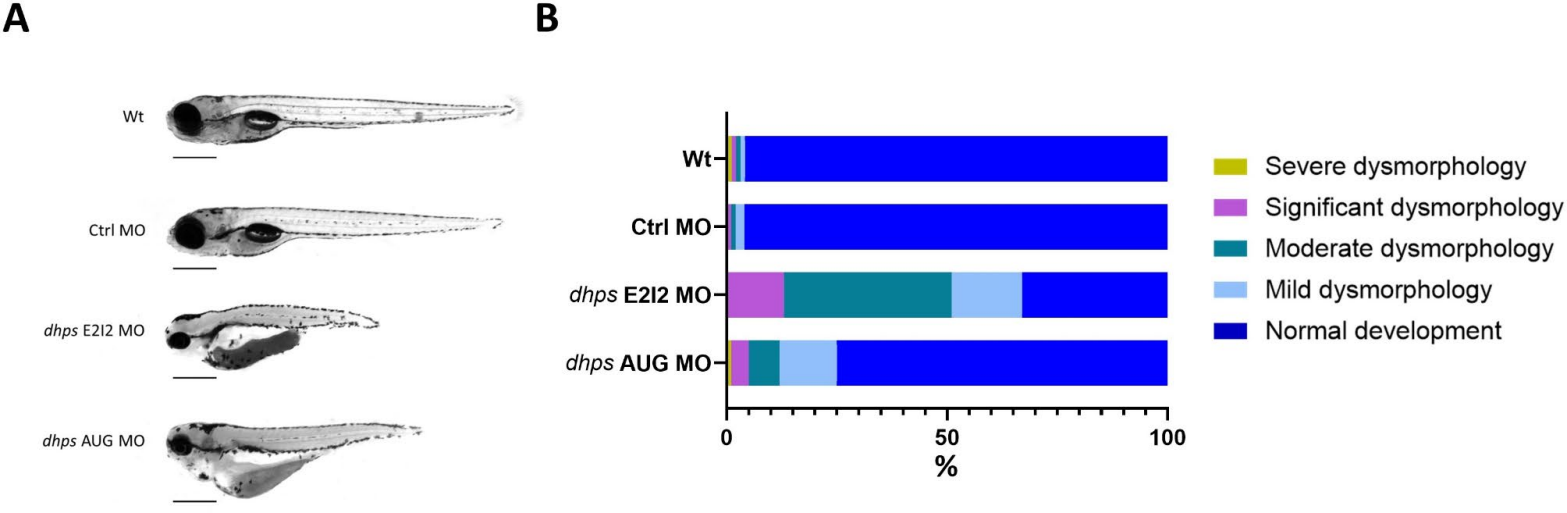
Phenotypic analysis of *dhps* knockdown zebrafish at five days post-fertilization (5 dpf). (**A**) Lateral view photographs of representative Wt, Ctrl MO, *dhps* E2I2 MO, and *dhps* AUG MO larvae, scale bar: 0.5 mm. (**B**) Chi-square analysis revealed significant differences between knockdown and control groups with regard to the percentages of larvae exhibiting normal development, mild and moderate dysmorphology (*p*<0.0001)

Phenotypic analysis of zebrafish larvae at 5 days post-fertilization (dpf), microinjected at one-cell stage with exon 2/intron 2 (E2I2) *dhps* E2I2 MO (7.92 ng) or with *dhps* AUG MO (11.55 ng), revealed that both antisense MOs caused similar dysmorphologies. *dhps* knockdown larvae were microcephalic with axis truncation, hyperpigmentation, cardiac edema, uninflated swim bladders, and body curvature, developmental delay and higher death rate at early stages compared to uninjected Wt and Ctrl MO (Fig. 2A). In control groups (Wt, *n* = 168 and Ctrl MO, *n* = 148), 96% of larvae exhibited normal development. In the *dhps* E2I2 MO group (*n* = 180), 13% of larvae exhibited significant dysmorphology, 38% moderate dysmorphology, and 16% mild dysmorphology. In the *dhps* AUG MO group (*n* = 126), 4% of larvae exhibited severe dysmorphology, 7% moderate dysmorphology, and 13% mild dysmorphology (Fig. 2B).

### Partial rescue of dysmorphology in *dhps* knockdown larvae by expression of *dhps* mRNA

**Fig. 3:**
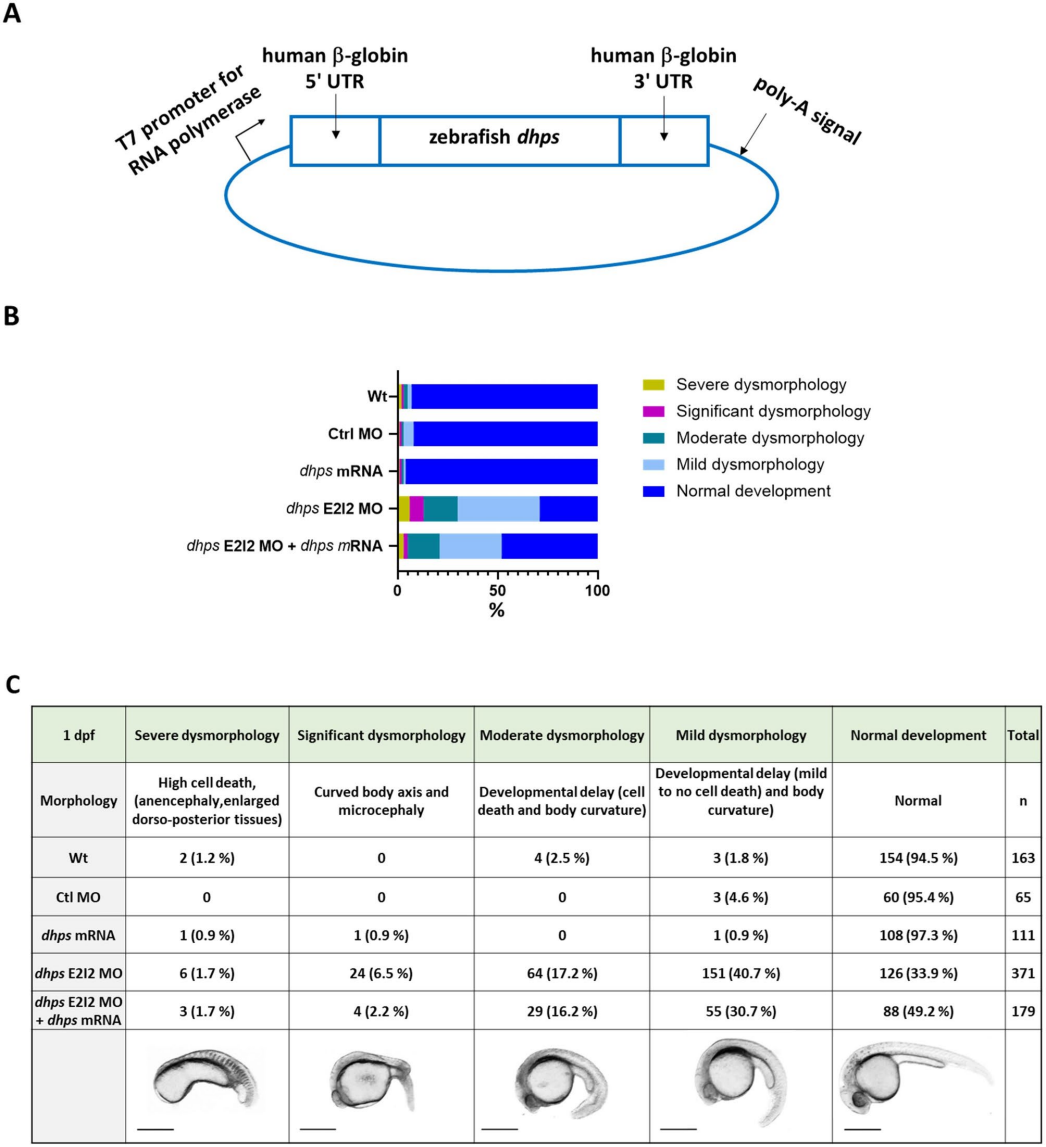
Partial rescue of *dhps* knockdown larvae by expression of *dhps* mRNA at 1 day post-fertilization (1 dpf). (**A**) Map of pIVT construct with zebrafish *dhps* cDNA. (**B**) mRNA overexpression partially shifted the more severe phenotypic categories of *dhps* knockdown to less severe categories. Chi-square analysis showed significant differences between control, knockdown and rescue groups with regard to the percentages of embryos exhibiting normal development, mild and moderate dysmorphology (*P*<0.0001). (**C**) Definition of observed phenotypic categories and sample size of each group

To control for antisense MO specificity, in vitro transcribed wild-type zebrafish *dhps* mRNA was co-injected with the *dhps* E2I2 MO to rescue the knockdown phenotype. Co-injection of in vitro transcribed *dhps* mRNA with *dhps* E2I2 MO reduced the number of larvae with dysmorphology and developmental delay, increasing in the percentage of normally developed larvae. In the *dhps* E2I2 MO group (*n* = 371), only 34% of embryos exhibited normal development, 41% mild dysmorphology, and 17% moderate dysmorphology, while in the group co-injected with *dhps* E2I2 MO and *dhps* mRNA (*n* = 179), 49% of embryos exhibited normal development, 31% mild dysmorphology, and 16% moderate dysmorphology. In control groups (Wt, *n* = 163 and *dhps* mRNA, *n* = 111) more than 94% of embryos exhibited normal development. These results demonstrate a partial rescue of the effects of *dhps* antisense MO (Fig. 3B, and C).

### Electrophysiological analysis of *dhps* knockdown zebrafish larvae

**Fig. 4:**
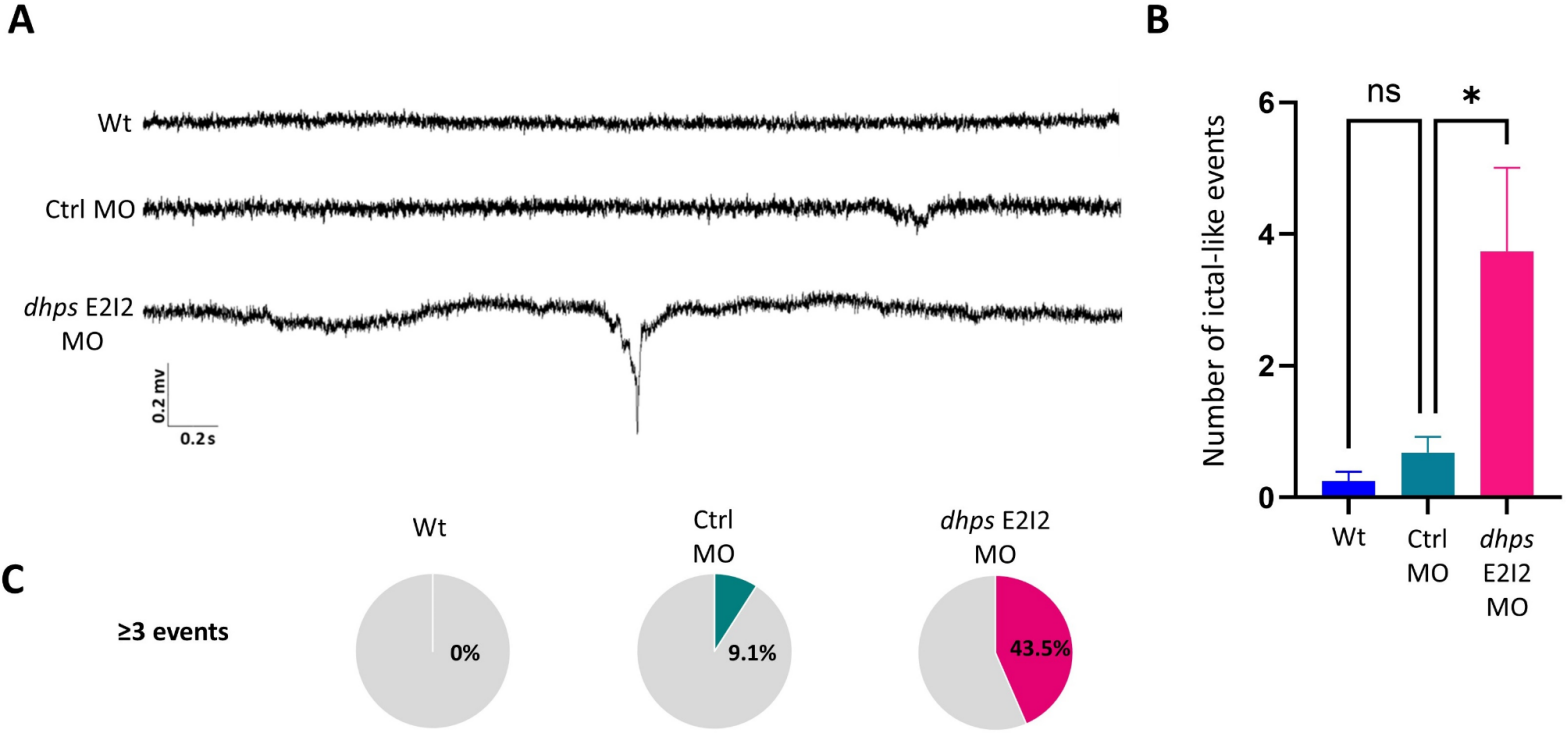
LFP recording of *dhps* knockdown larvae. (**A**) Snapshot of LFP signal recorded from 4-dpf Wt, Ctrl MO and *dhps* E2I2 MO larvae. (**B**) One-way ANOVA revealed that the frequency of ictal-like events in *dhps* knockdown larvae was significantly increased compared to control larvae (*p* ≤ 0.02). Each recording was performed over a period of 20 min. (**C**) Each pie chart shows percentage of larvae with more than 3 ictal-like events during 20 min of recording. While in Ctrl MO larvae less than 10% of larvae had more than 3 ictal-like events, in *dhps* E2I2 MO-injected larvae, more than 43% of larvae had more than 3 ictal-like events

Local field potential (LFP) recordings from the optic tectum revealed epileptiform events in 4-dpf *dhps* knockdown larvae. LFP recording of brain activity of 4-dpf *dhps* knockdown larvae revealed spontaneous electrographic discharges with high amplitude (*≥* three-fold baseline) versus controls (Fig. 4A). Quantification of ictal-like events revealed that uninjected Wt larvae (*n* = 20) had a mean of 0.25 ± 0.14(SEM), Ctrl MO larvae (*n* = 22) had mean of 0.68 ± 0.24(SEM) and *dhps* E2I2 MO larvae (*n* = 23) had a mean of 3.74 ± 1.27(SEM). A significant increase was observed in the *dhps* knockdown group using the one-way ANOVA test (*p* ≤ 0.02) (Fig. 4B).

### Reduced GABAergic neuronal arborization in *dhps* knockdown larvae

**Fig. 5:**
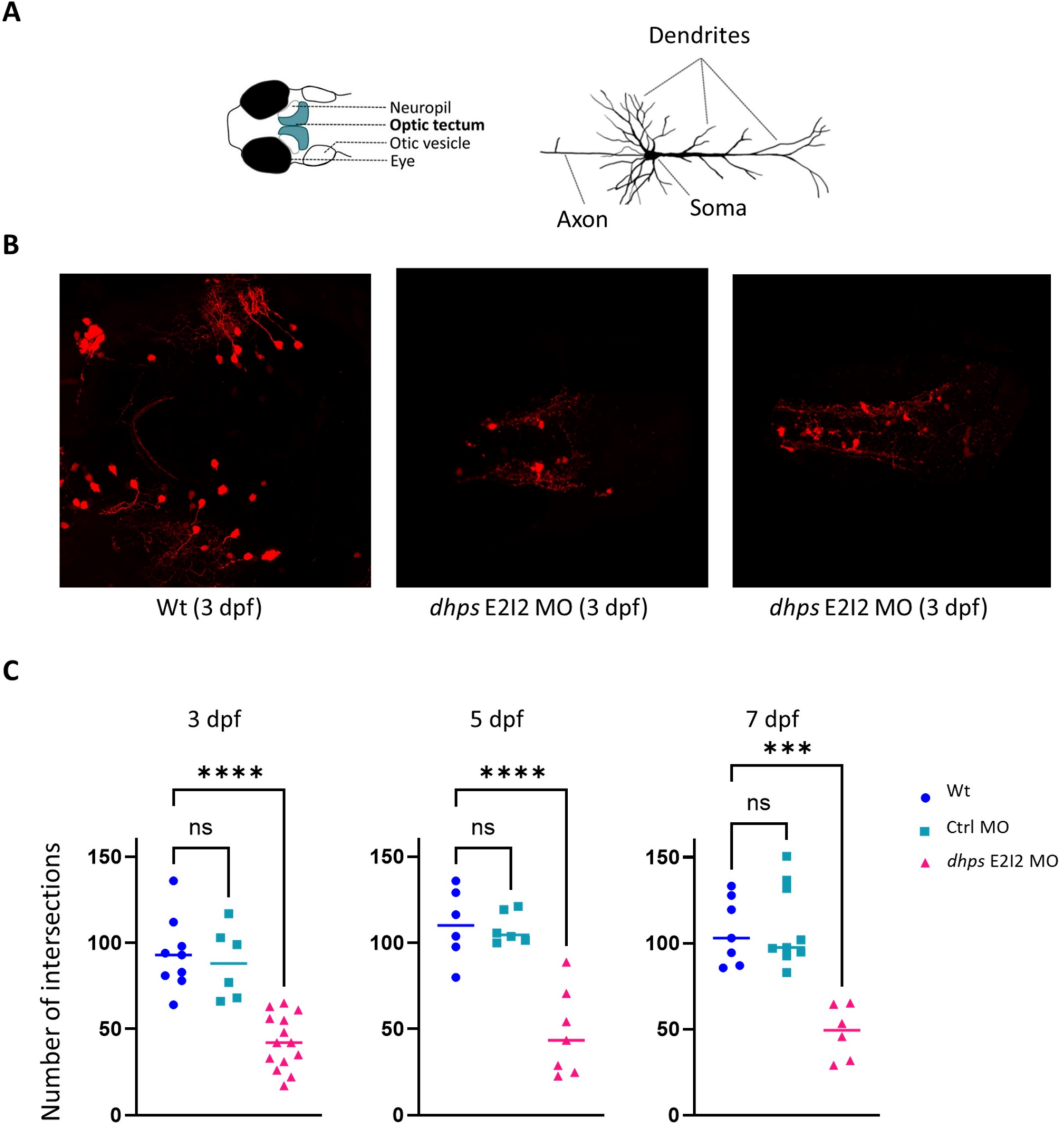
GABAergic neuronal dendritic arborization. (**A**) Larval head with location of optic tectum (blue) and neuropil (light yellow) where that GABAergic neurons project their arbors. (**B**) Confocal microscopy revealed that *dhps* knockdown larvae have less complex dendritic arborization in the GABAergic neurons. (**C**) GABAergic neuronal arbors were quantified in zebrafish larvae at three different ages (3, 5, and 7 dpf). At all three developmental ages, via one way ANOVA there was a significant reduction in the number of neuronal arbors in the *dhps* knockdown larvae (*dhps* E2I2 MO) versus both wild-type control larvae (Wt) and control MO-injected larvae (Crtl MO) (see also Table 1 below)

Confocal microscopy analysis of *dhps* knockdown larvae derived from a transgenic reporter line with GABAergic-specific expression of mCherry assessed the effects of reduced *dhps* function on GABAergic neuron development. In zebrafish larvae, GABAergic neurons project arbors from the optic tectum to the tectal neuropil (Fig. 5A). Confocal microscopy revealed significantly reduced arborization of GABAergic neurons in *dhps* knockdown larvae (Fig. 5B). Quantification of the GABAergic neuronal arborization by Sholl analysis revealed a decreased dendritic arborization (p<0.0001, Table 1) in larvae with reduced *dhps* expression (Fig. 5C).

**Table 1:** Quantification of the number of neuronal arbors in wild-type control larvae (wt), control MO-injected larvae (Cntl MO), and *dhps* knockdown larvae (*dhps* E2I2 MO) at 3, 5 and 7 dpf

Age	3 dpf			5 dpf			7 dpf		
Group	Wt	Ctrl Mo	*dhps* E212 MO	Wt	Ctrl Mo	*dhps* E212 MO	Wt	Ctrl Mo	*dhps* E212 MO
n	7	6	14	6	6	7	7	9	6
Mean	93.22	88.33	42.57	110.4	108.3	47.55	107.3	109.6	48.19
SEM	7.01	8.54	4.23	8.5	3.8	9.49	7.41	7.89	6.38

## Discussion

Our zebrafish loss-of-function model of DHPS deficiency by antisense knockdown of *dhps* expression mimicked features of the human DHPS deficiency syndrome. The zebrafish showed developmental delayed with epileptiform discharges, as well as microcephaly, axis truncation, and body curvature. Electrophysiological analysis of *dhps* knockdown larvae showed increased epileptiform activity, while confocal microscopy analysis revealed significantly reduced arborisation and complexity in GABAergic neurons.

The epileptiform discharges in *dhps* knockdown larvae paralleled the epileptiform activity on electroencephalography (EEG) and seizures in DHPS deficiency syndrome patients [20], and in mice with a brain-specific knockout of *Dhps* induced by postnatal intraventricular injection of a Cre-expressing AAV vector [3]. Seizures were not reported in patients with loss-of-function *EIF5A* mutations [18], nor in an eIF5A-deficient zebrafish model [18, 25]. Patients with *DOHH* mutations have seizures [19]. Our findings suggest that for the seizure phenotype, the brain is more sensitive to reduced eIF5A hypusination caused by mutations in *DHPS* or *DOHH*, while patients with neurodevelopmental disorders caused by autosomal dominant *EIF5A* mutations do not exhibit seizures as remaining eIF5A^HYP^ levels may be sufficient to prevent epileptiform activity and seizures.

Confocal microscopy analysis of *dhps* knockdown zebrafish using a transgenic reporter line revealed significantly reduced GABAergic neuron aborization. Loss of inhibitory GABAergic inputs may contribute to neuronal hyperexcitability, but mouse model studies are needed to confirm these findings. Moreover, cell type analyses in knockdown zebrafish brains could determine if this phenotype results from overall neuronal loss or a selective loss of GABAergic neurons. Brain magnetic resonance imaging scans of DHPS deficient patients are normal [OD et al., unreported data], suggesting that the brain morphology phenotypes in our zebrafish and other mouse models [3, 24] are more pronounced than the human phenotype.

Since hypusination of eIF5A is a key regulator of autophagy [14, 27], and autophagy is critical during axonal and presynaptic development [28], impaired autophagy during zebrafish and mouse brain development in the DHPS deficiency syndrome models should be explored as a pathogenic mechanism. Our analysis of protein expression in *dhps* knockdown zebrafish reveal a striking reduction in expression of larger proteins. Ongoing studies using proteomic and transcriptomic analysis of embryonic brains with reduced hypusination beginning during early development may identify key molecular and cellular changes at different developmental stages and may elucidate the role of eIF5A^HYP^ in the translation of certain proteins (e.g., long polypeptides [10] and neurosecretory factors [5]). We need to better understand the reductions in total eIF5A and eIF5A^HYP^ levels seen in *dhps* knockdown zebrafish, which are similar to those in conditional cell-specific mouse models of DHPS loss [4, 5].

Overexpression of *dhps* mRNA in wild-type embryos did not cause a deleterious phenotype, suggesting that gene therapy strategies that involve overexpression of DHPS may show benefit for DHPS deficiency syndrome patients. Conversely, only partial rescue of the *dhps* knockdown phenotype was achieved by overexpression of *dhps* mRNA, which may be due to the limited half-life of *dhps* mRNA after microinjection at the single-cell stage.

Our overall findings support that hypusination of eIF5A is important for early brain development, and zebrafish with reduced *dhps* expression are a useful model for DHPS deficiency syndrome. Future experiments with this model will evaluate anti-seizure medications using the seizure phenotype, as well as other therapeutic modalities and endpoints. Generation of zebrafish models of neurodevelopmental disorders caused by mutations in the DHPS-DOHH-eIF5A pathway that incorporate specific patient mutations into the genetically engineered lines could dissect the phenotypic differences observed between these related diseases.

## Methods

### Zebrafish husbandry

Wild-type (Wt) adult zebrafish (*Danio rerio*; AB strain; CVE-KIT) were maintained at 28.5 °C on a 14-h/10-h light/dark cycle under standard aquaculture conditions, and fertilized eggs were collected via natural spawning. Embryos were raised in embryo medium (E3; 1.5 mmol/L HEPES, pH 7.6, 17.4 mmol/L NaCl, 0.21 mmol/L KCl, 0.12 mmol/L MgSO4, and 0.18 mmol/L Ca[NO3]2), under the same conditions as adults. For all zebrafish experiments conducted at NCMM, larvae up to 7 days post-fertilization (dpf) were used.

### Antisense morpholino oligomers (MOs) and microinjections

11.55 ng of a translation-blocking MO (*dhps* AUG MO: 5’ GGTTATGGATGTAAATCCGGCTTTT) targeting the AUG start codon of the zebrafish *dhps* mRNA and 7.92 ng of splice site-blocking MO targeting the exon 2/intron 2 splice site of the zebrafish *dhps* pre-RNA (*dhps* E2I2 MO: 5′ CACGATCAGTCTGTCACTCACCATC) were used to achieve partial knockdown of zebrafish *dhps*. Fluoresceinated standard control MO was used as a negative control (Ctrl MO) (11.55 and 7.92 ng respectively). MOs were designed and synthesized by Gene Tools LLC (Philomath, Oregon, USA) and injected into 1-2-cell stage embryos [26].

### Reverse transcriptase PCR (RT-PCR)

Efficiency of knockdown was determined by RT-PCR, using primers that amplify across the predicted deletion: 5′ GCGCTGTGAAATGTGAGTGAAAC and 5′ GTTTGACGTGTAGCCCAGGAAT. The PCR amplicon was 385 bp in the control embryos and 172 bp in the *dhps* MO-injected embryos, and was visualized by standard agarose gel electrophoresis [26].

### Western blot analysis

Zebrafish embryos were evaluated by Western blot analysis, adapting methods previously described [3]. Specifically, 30–40 zebrafish embryos (2 dpf) were lysed in 400 µL of buffer containing 50 mM Tris, pH 8.0, 150 mM NaCl, 0.05% deoxycholate, 0.1% IGEPAL CA-630, 0.1% SDS, 0.2% sarcosyl, 10% glycerol, 1 mM DTT, 1 mM EDTA, 10mM NaF, protease inhibitors (#11836170001, Roche), phosphatase inhibitors (#4906845001, Roche), 2 mM MgCl2, and 0.05% v/v Benzonase (Millipore) and were intermittently vortexed to facilitate protein extraction. Protein was quantified using the DC Protein Assay Kit II (#5000112, Bio-Rad) followed by SDS-PAGE (4–20% gel). Separated protein (20 µg) was transferred to PVDF membranes and blocked in Odyssey Blocking Buffer (#927-40100, LI-COR Biosciences) at room temperature for 1 h. Membranes were incubated with REVERT (#926-11016; LI-COR Biosciences) to permit visualization of total protein. Subsequent incubation with primary antibodies diluted in Intercept Blocking Buffer (#927-70001; LI-COR Biosciences) was performed overnight at 4 °C. Membranes were washed twice with TBST buffer prior to incubation with near infrared, fluorescent dye-conjugated secondary antibodies at room temperature for 1 h. Following additional washes with TBST buffer, the membranes were imaged using an Odyssey CLx Imaging System and images were analyzed using the CLx Image Studio Version 5.2 Software (LI-COR Biosciences).

The following primary antibodies were used at the dilutions indicated: mouse anti-deoxyhypusine synthase (1:2000; Santa Cruz, #sc-365077), mouse anti-eIF5A^TOTAL^ (1:2000; BD Biosciences, #611977), and rabbit anti-eIF5A^HYP^ (1:5000; Millipore, #ABS1064-I). Densitometric data are graphed as relative expression.

### mRNA rescue

To generate *dhps* RNA, zebrafish *dhps* cDNA was cloned into the pIVT expression construct [Addgene plasmid 122139; 29], which was linearized through restriction at the 3’ end of the ORF and used as a template to generate *dhps* mRNA using T7 RNA polymerase in an in vitro transcription reaction.

### Local field potential (LFP) recording

Recordings were obtained from tecta at 4 dpf [30]. Seizure detection was performed through visual inspection and automated using a custom-written R script to minimize bias and artifacts due to muscle contractions. Recorded frequencies were divided 1-100, 100–250, and 250–500 Hz bands. If amplitude exceeded 3× background, the event was considered a seizure based on high-frequency oscillations (> 100 Hz) [31]. Power spectrum was analyzed using Clampfit 10.7 software (Molecular Devices). 20-minute-long recordings were used to compute the power spectrum from larvae at 4 dpf, and each condition was averaged per group [32].

### Confocal microscopy and quantification of arbors

To visualize GABAergic neurons, wild-type and *dhps* E2I2 MO-microinjected embryos were treated from 1 dpf with 0.003% phenylthiourea to prevent pigmentation. Larvae were anesthetized in 0.001% tricaine (Sigma), fixed for 3 h at room temperature with 4% paraformaldehyde, mounted on glass slides, and imaged using confocal microscopy. A dorsal z-stack of the optic tectum was collected using a 40x lens and a z-resolution of 0.44 μm. For Sholl analysis [33, 34], images were filtered using the 3D-Median filter in ImageJ. A z-projection of the tectum was generated, and the resulting image was converted to a thresholded binary image. Arborization extent was quantified using Sholl analysis (plug-in; http://imagej.net/Sholl_Analysis). The number of intersections was normalized against the number of neurons in the imaged area; this value was statistically analyzed [32].

## Data Availability

The original contributions presented in the study are included in the article/supplementary material, further inquiries can be directed to the corresponding author.

## Abbreviations

DHPS: Deoxyhypusine synthase
DOHH: Deoxyhypusine hydroxylase
E2I2: Exon 2/intron 2
EEG: Electroencephalography
eIF5A: Eukaryotic initiation factor 5 A
eIF5A^HYP^: Hypusinated eIF5A
HIF1A: Hypoxia-inducible factor 1-alpha
LFP: Local field potential
MO: Antisense morpholino oligomer
NDD: Neurodevelopmental disorder
NMD: Nonsense-mediated decay
SUMO: Small ubiquitin-like modifier
RT-PCR: Reverse transcriptase PCR
SEM: Standard error of the mean
Wt: Wild-type

## References

[1] Park MH, Wolff EC. Hypusine, a polyamine-derived amino acid critical for eukaryotic translation. J Biol Chem. 2018. 10.1074/jbc.TM118.003341. 30257869 10.1074/jbc.TM118.003341 PMC6290153

[2] Zhang Y, Su D, Zhu J, et al. Oxygen level regulates N-terminal translation elongation of selected proteins through deoxyhypusine hydroxylation. Cell Rep. 2022. 10.1016/j.celrep.2022.110855. 35613595 10.1016/j.celrep.2022.110855 PMC9218932

[3] Padgett LR, Shinkle MR, Rosario S, et al. Deoxyhypusine synthase mutations alter the post-translational modification of eukaryotic initiation factor 5A resulting in impaired human and mouse neural homeostasis. HGG Adv. 2023. 10.1016/j.xhgg.2023.100206. 37333770 10.1016/j.xhgg.2023.100206 PMC10275725

[4] Padgett LR, Robertson MA, Anderson-Baucum EK, et al. Deoxyhypusine synthase, an essential enzyme for hypusine biosynthesis, is required for proper exocrine pancreas development. FASEB J. 2021. 10.1096/fj.201903177R. 33811703 10.1096/fj.201903177R PMC8034418

[5] Connors CT, Villaca CBP, Anderson-Baucum EK, et al. A translational regulatory mechanism mediated by hypusinated eukaryotic initiation factor 5A facilitates β-cell identity and function. Diabetes. 2024. 10.2337/db23-0148. 38055903 10.2337/db23-0148 PMC10882153

[6] Schuller AP, Wu CC, Dever TE, Buskirk AR, Green R. eIF5A functions globally in translation elongation and termination. Mol Cell. 2017. 10.1016/j.molcel.2017.03.003. 28392174 10.1016/j.molcel.2017.03.003 PMC5414311

[7] Pelechano V, Alepuz P. eIF5A facilitates translation termination globally and promotes the elongation of many non polyproline-specific tripeptide sequences. Nucleic Acids Res. 2017. 10.1093/nar/gkx479. 28549188 10.1093/nar/gkx479 PMC5499558

[8] Gutierrez E, Shin BS, Woolstenhulme CJ, et al. eIF5A promotes translation of polyproline motifs. Mol Cell. 2013. 10.1016/j.molcel.2013.04.021. 23727016 10.1016/j.molcel.2013.04.021 PMC3744875

[9] Hoque M, Park JY, Chang YJ, et al. Regulation of gene expression by translation factor eIF5A: Hypusine-modified eIF5A enhances nonsense-mediated mRNA decay in human cells. Translation (Austin). 2017. 10.1080/21690731.2017.1366294. 29034140 10.1080/21690731.2017.1366294 PMC5630042

[10] Abe T, Nagai R, Shimazaki S, et al. In vitro yeast reconstituted translation system reveals function of eIF5A for synthesis of long polypeptide. J Biochem. 2020. 10.1093/jb/mvaa022. 32053170 10.1093/jb/mvaa022

[11] Li T, Belda-Palazón B, Ferrando A, Alepuz P. Fertility and polarized cell growth depends on eIF5A for translation of polyproline-rich formins in Saccharomyces cerevisiae. Genetics. 2014. 10.1534/genetics.114.166926. 24923804 10.1534/genetics.114.166926 PMC4125393

[12] Pálfi P P, Bakacsy L, Kovács H, Szepesi Á Á. Hypusination, a metabolic posttranslational modification of eIF5A in plants during development and environmental stress responses. Plants. 2021. 10.3390/plants1007126. 34206171 10.3390/plants10071261 PMC8309165

[13] Liang Y, Piao C, Beuschel CB, et al. eIF5A hypusination, boosted by dietary spermidine, protects from premature brain aging and mitochondrial dysfunction. Cell Rep. 2021. 10.1016/j.celrep.2021.108941. 33852845 10.1016/j.celrep.2021.108941

[14] Hofer SJ, Liang Y, Zimmermann A, et al. Spermidine-induced hypusination preserves mitochondrial and cognitive function during aging. Autophagy. 2021. 10.1080/15548627.2021.1933299. 34105442 10.1080/15548627.2021.1933299 PMC8386697

[15] Puleston DJ, Baixauli F, Sanin DE, et al. Polyamine metabolism is a central determinant of helper T cell lineage fidelity. Cell. 2021. 10.1016/j.cell.2021.06.007. 34216540 10.1016/j.cell.2021.06.007 PMC8358979

[16] Anderson-Baucum E, Piñeros AR, Kulkarni A, et al. Deoxyhypusine synthase promotes a pro-inflammatory macrophage phenotype. Cell Metabol. 2021. 10.1016/j.cmet.2021.08.003. 10.1016/j.cmet.2021.08.003 PMC8432737 34496231

[17] Sfakianos AP, Raven RM, Willis AE. The pleiotropic roles of eIF5A in cellular life and its therapeutic potential in cancer. Biochem Soc Trans. 2022. 10.1042/BST20221035. 36511302 10.1042/BST20221035 PMC9788402

[18] Faundes V, Jennings MD, Crilly S, et al. Impaired eIF5A function causes a mendelian disorder that is partially rescued in model systems by spermidine. Nat Commun. 2021. 10.1038/s41467-021-21053-2. 33547280 10.1038/s41467-021-21053-2 PMC7864902

[19] Ziegler A, Steindl K, Hanner AS, et al. Bi-allelic variants in DOHH, catalyzing the last step of hypusine biosynthesis, are associated with a neurodevelopmental disorder. Am J Hum Genet. 2022. 10.1016/j.ajhg.2022.06.010. 35858628 10.1016/j.ajhg.2022.06.010 PMC9388783

[20] Ganapathi M, Padgett LR, Yamada K, et al. Recessive rare variants in Deoxyhypusine synthase, an enzyme involved in the synthesis of Hypusine, are Associated with a neurodevelopmental disorder. Am J Hum Genet. 2019. 10.1016/j.ajhg.2018.12.017. 30661771 10.1016/j.ajhg.2018.12.017 PMC6369575

[21] Templin AT, Maier B, Nishiki Y, Tersey SA, Mirmira RG. Deoxyhypusine synthase haploinsufficiency attenuates acute cytokine signaling. Cell Cycle. 2011. 10.4161/cc.10.7.15206. 21389784 10.4161/cc.10.7.15206 PMC3100881

[22] Nishimura K, Lee SB, Park JH, Park MH. Essential role of eIF5A-1 and deoxyhypusine synthase in mouse embryonic development. Amino Acids. 2012. 10.1007/s00726-011-0986-z. 21850436 10.1007/s00726-011-0986-z PMC3220921

[23] Sievert H, Pällmann N, Miller KK, et al. A novel mouse model for inhibition of DOHH-mediated hypusine modification reveals a crucial function in embryonic development, proliferation and oncogenic transformation. Dis Model Mech. 2014. 10.1242/dmm.014449. 24832488 10.1242/dmm.014449 PMC4107325

[24] Kar RK, Hanner AS, Starost MF, et al. Neuron-specific ablation of eIF5A or deoxyhypusine synthase leads to impairments in growth, viability, neurodevelopment, and cognitive functions in mice. J Biol Chem. 2021. 10.1016/j.jbc.2021.101333. 34688659 10.1016/j.jbc.2021.101333 PMC8605248

[25] Carvalho CM, Vasanth S, Shinawi M, et al. Dosage changes of a segment at 17p13.1 lead to intellectual disability and microcephaly as a result of complex genetic interaction of multiple genes. Am J Hum Genet. 2014. 10.1016/j.ajhg.2014.10.006. 25439725 10.1016/j.ajhg.2014.10.006 PMC4225592

[26] Mastracci TL, Robertson MA, Mirmira RG, Anderson RM. Polyamine biosynthesis is critical for growth and differentiation of the pancreas. Sci Rep. 2015. 10.1038/srep13269. 26299433 10.1038/srep13269 PMC4547391

[27] Gobert AP, Finley JL, Latour YL, et al. Hypusination orchestrates the Antimicrobial response of macrophages. Cell Rep. 2020. 10.1016/j.celrep.2020.108510. 33326776 10.1016/j.celrep.2020.108510 PMC7812972

[28] Crawley O, Grill B. Autophagy in axonal and presynaptic development. Curr Opin Neurobiol. 2021. 10.1016/j.conb.2021.03.011. 33940492 10.1016/j.conb.2021.03.011 PMC8387345

[29] Igarashi H, Knott JG, Schultz RM, Williams CJ. Alterations of PLCbeta1 in mouse eggs change calcium oscillatory behavior following fertilization. Dev Biol. 2007. 10.1016/j.ydbio.2007.09.028. 17961538 10.1016/j.ydbio.2007.09.028 PMC2170533

[30] Afrikanova T, Serruys AS, Buenafe OE, et al. Validation of the zebrafish pentylenetetrazol seizure model: locomotor versus electrographic responses to antiepileptic drugs. PLoS ONE. 2013. 10.1371/journal.pone.0054166. 23342097 10.1371/journal.pone.0054166 PMC3544809

[31] Zijlmans M, Jiruska P, Zelmann R, Leijten FS, Jefferys JG, Gotman J. High-frequency oscillations as a new biomarker in epilepsy. Ann Neurol. 2012. 10.1002/ana.22548. 22367988 10.1002/ana.22548 PMC3754947

[32] Tiraboschi E, Martina S, van der Ent W, et al. New insights into the early mechanisms of epileptogenesis in a zebrafish model of Dravet syndrome. Epilepsia. 2020. 10.1111/epi.16456. 32096222 10.1111/epi.16456

[33] Ferreira TA, Blackman AV, Oyrer J, et al. Neuronal morphometry directly from bitmap images. Nat Methods. 2014. 10.1038/nmeth.3125. 25264773 10.1038/nmeth.3125 PMC5271921

[34] Ristanović D, Milosević NT, Stulić V. Application of modified Sholl analysis to neuronal dendritic arborization of the cat spinal cord. J Neurosci Methods. 2006. 10.1016/j.jneumeth.2006.05.030. 16814868 10.1016/j.jneumeth.2006.05.030

